# Supplementary material for: PyRINEX: a new multi-purpose Python package for GNSS RINEX data
Source: PeerJ Comput Sci. 2024 Jan 16;10:e1800. doi: 10.7717/peerj-cs.1800 (PMC10803049; doi:10.7717/peerj-cs.1800)
Supplement: Supplemental Information 1 [file peerj-cs-10-1800-s001.pdf]

Methods of calculating GPS satellite positions

The methodology note on GPS satellite position calculation is from https://zhuanlan.zhihu.com/p/435129629.

(1)Calculate the average angular velocity n

$$n0 = \sqrt{u/a^3}$$

where the long semi-axis a of the satellite orbit is given by the broadcast ephemeris and u is the Earth's gravitational constant (u = GM where G is the universal gravitational constant and M is the mass of the Earth)

The satellite mean angular velocity n is then derived from the ingress param eter Δn given by the ephemeris.

$$n = n0 + \Delta n$$

(2)Calculate the angle of the satellite's flat perihelion at the instant of observation M.

$$M = M0 + n(t - TOE)$$

The reference moment TOE and the horizon angle M0 corresponding to the reference TOE moment are given by the broadcast ephemeris. Here we need to note that the receiver receives the satellite signal moment in the observation file, and for the UTC. need to be converted to GPS week + seconds of the week format and the ephemeris file within the TOE subtracted from the regularization time.

(3)Calculate the angle of the off-set point E

$$E_k = M + e\sin\ E_{k1}$$

Solve Kepler's equations iteratively to find the declination angle E, where e the eccentricity of the satellite orbit and M the angle of the flat declination is given by the broadcast ephemeris.

(4)Calculate the true proximal angle f.

$$\tan f/2 = \sqrt{1 + e/1_e}\tan\ E/2$$

In order to easily determine the quadrant in which the true proximal angle f appears when solving inverse trigonometric functions, the half-angle formula is used.

(5)Calculate the ascending angle of separation θ'

$$\theta' = f + \omega$$

Where ω is the perigee amplitude angle, given by the broadcast ephemeris.

(6)Calculate the ingress correction terms

$$\sigma_\theta = C_{\theta c}\cos\ 2\theta' + C_{\theta s}\sin\ 2\theta'$$
$$\sigma_\gamma = C_{\gamma c}\cos\ 2\theta' + C_{\gamma s}\sin\ 2\theta'$$
$$\sigma_i = C_{ic}\cos\ 2\theta' + C_{is}\sin\ 2\theta'$$

Six of the regency correction parameters are given by the broadcast ephemeris.

(7) Perform regression correction

$$\theta = \theta' + \sigma_\theta$$
$$\gamma = \gamma' + \sigma_\gamma$$
$$i = i0 + \sigma_i + di/dt(t - TOE)$$

Use the regression correction parameters from the previous step to perform regression correction.

(8) Find the coordinates of the satellite in the orbital plane

$$x = \gamma\cos\,\theta$$
$$y = \gamma\sin\,\theta$$

(9) Calculate the longitude of the ascending node at the instant of observation

$$L = \Omega_0 + (\Omega. - we)t - \Omega.\cdot TOE$$

where Ω. is the ingress parameter, we is the angular velocity of the Earth's rotation, and t is the calendar element of the signal received by the receiver.

(10) Calculate the instantaneous position of the satellite

$$x' = x\cos\,L - y\cos\,i\cdot\sin\,L$$
$$y' = x\sin\,L + y\cos\,i\cdot\cos\,L$$
$$z' = y\sin\,i$$

Through the previous process to find the orbit surface inclination i, instantaneous ascending node longitude L, satellite in the orbit plane coordinates x,y to find the satellite in the instantaneous geosynchronous system in the three-dimensional coordinates x' , y' , z'.
