# Supplementary material for: PyRINEX: a new multi-purpose Python package for GNSS RINEX data
Source: PeerJ Comput Sci. 2024 Jan 16;10:e1800. doi: 10.7717/peerj-cs.1800 (PMC10803049; doi:10.7717/peerj-cs.1800)
Supplement: Supplemental Information 2 [file peerj-cs-10-1800-s002.pdf]

Running speed test of PyRINEX

This running speed test uses AMD Ryzen Threadripper 2970WX 24-Core Processor, 3000 Mhz, 24 cores, 48 logical processors. Timing using Python's time library.

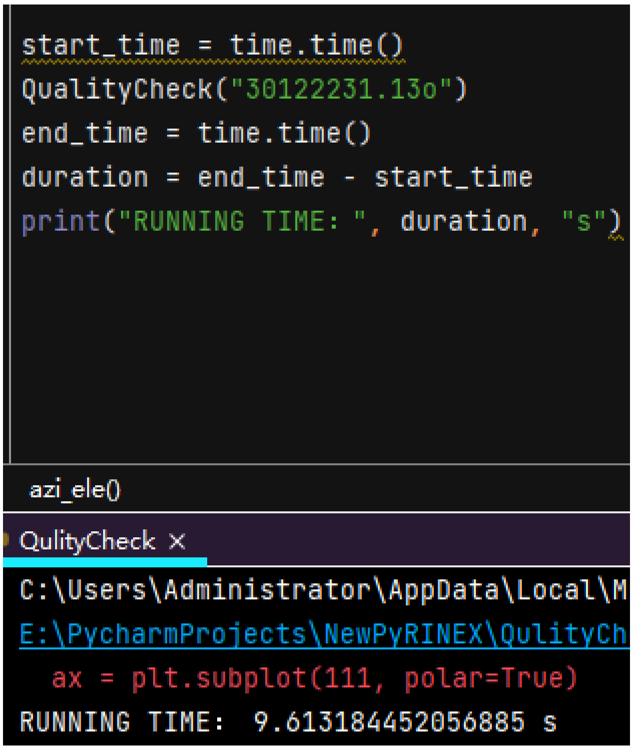

When processing a RINEX data with eight hours of data recorded internally, the quality check would take about nine seconds.

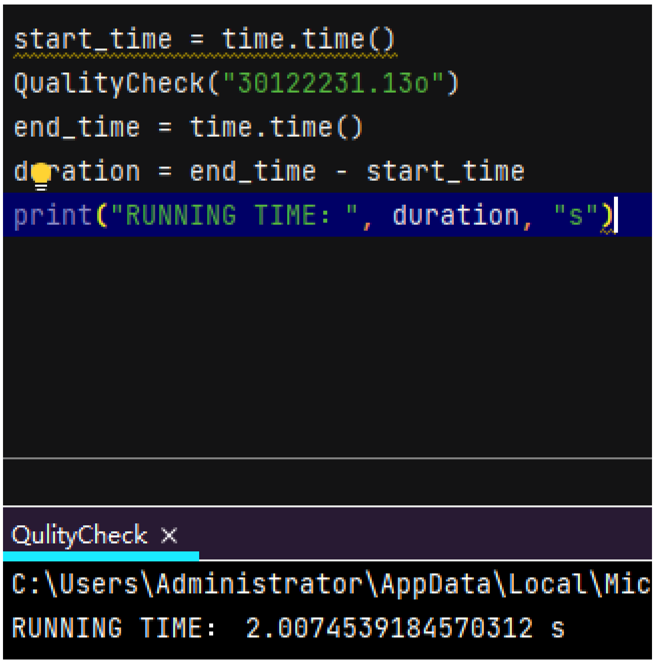

But if you choose not to output CSV files and schematics, it only takes about 2 seconds.

```
     3.02           OBSERVATION DATA    Mixed(MIXED)        RINEX VERSION / TYPE
cnvtToRINEX 3.14.0  convertToRINEX OPR  20210907 040721 UTC PGM / RUN BY / DATE
----------------------------------------------------------- COMMENT
XBN9A                                                       MARKER NAME
                                                            MARKER NUMBER
GEODETIC                                                    MARKER TYPE
```

```
     2.10           OBSERVATION DATA    G (GPS)             RINEX VERSION / TYPE
Pinnacle 1.00                           16-NOV-08 16:01     PGM / RUN BY / DATE
build April 19, 2002                                        COMMENT
-Unknown-           -Unknown-                               OBSERVER / AGENCY
         Begin Pinnacle project                             COMMENT
1115                                                        COMMENT
                                                            COMMENT
                                                            COMMENT
[illegible]
```

We tested different versions of RINEX data for quality checks and found that they all worked properly.
