# Supplementary figures and images for: PyRINEX: a new multi-purpose Python package for GNSS RINEX data
Source: PeerJ Comput Sci. 2024 Jan 16;10:e1800. doi: 10.7717/peerj-cs.1800 (PMC10803049; doi:10.7717/peerj-cs.1800)

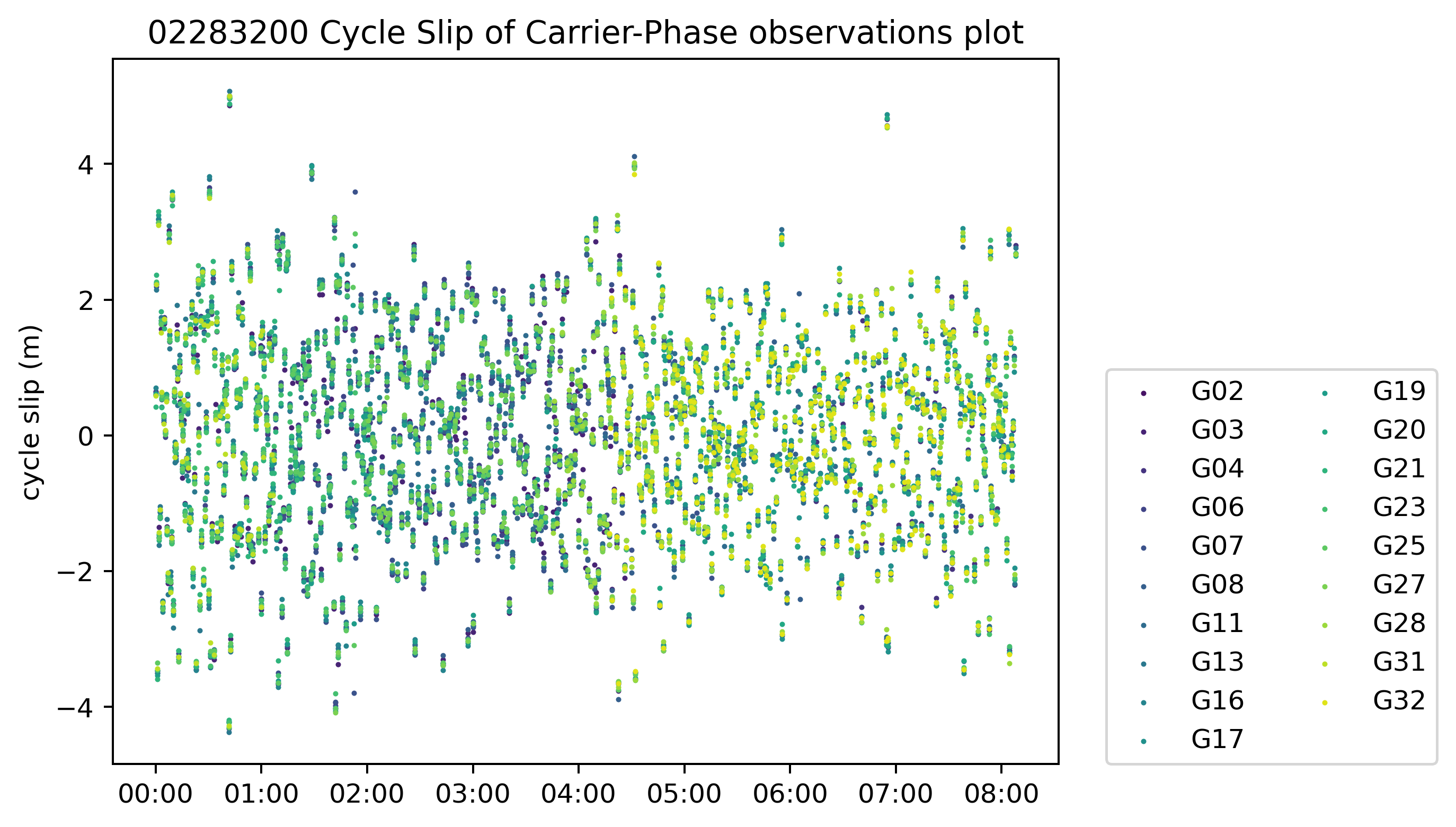

Supplement: Supplemental Information 3 [file peerj-cs-10-1800-s003.zip › 02283200CycleSlipCarrier.png]

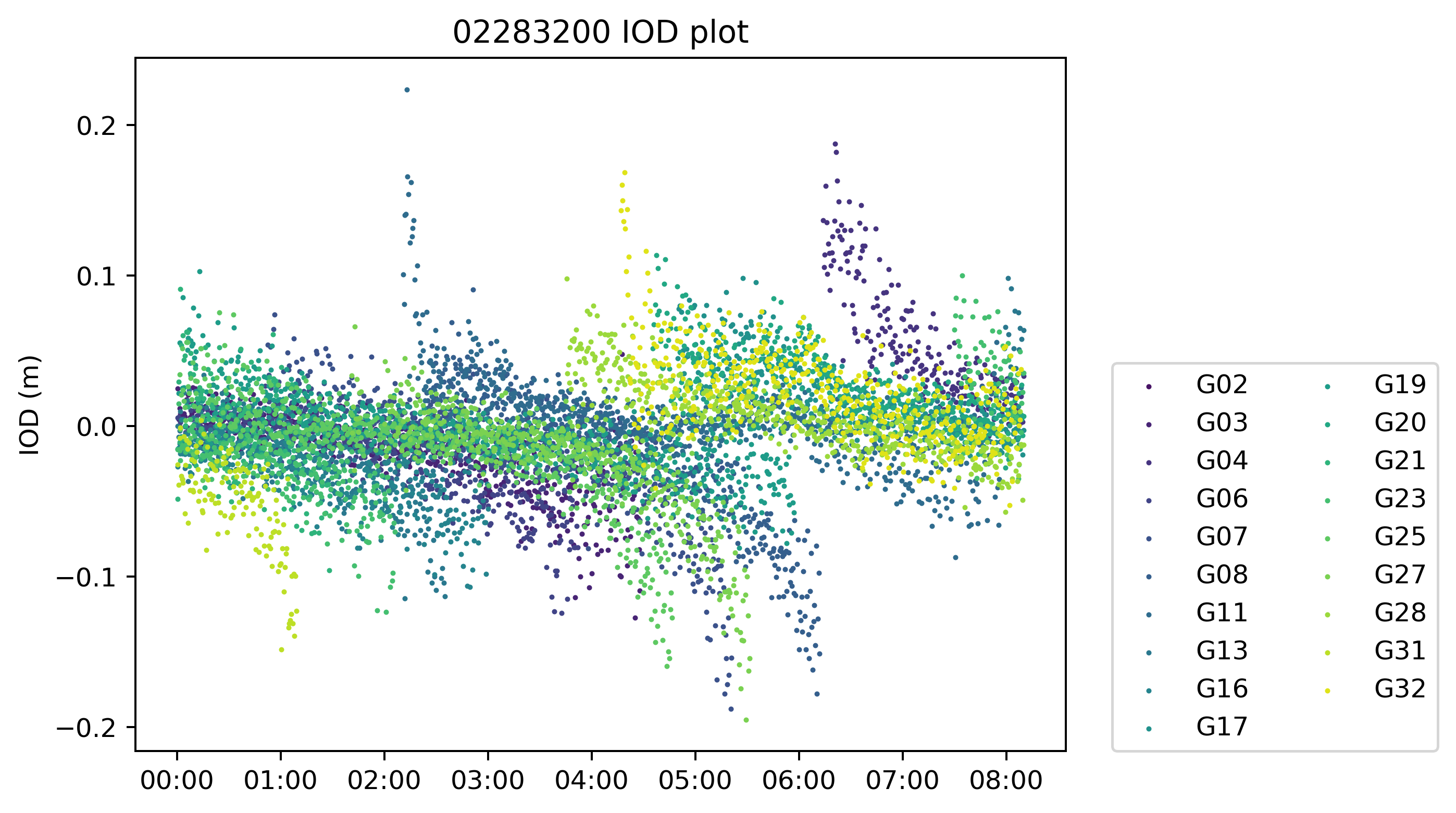

Supplement: Supplemental Information 3 [file peerj-cs-10-1800-s003.zip › 02283200IOD_plot.png]

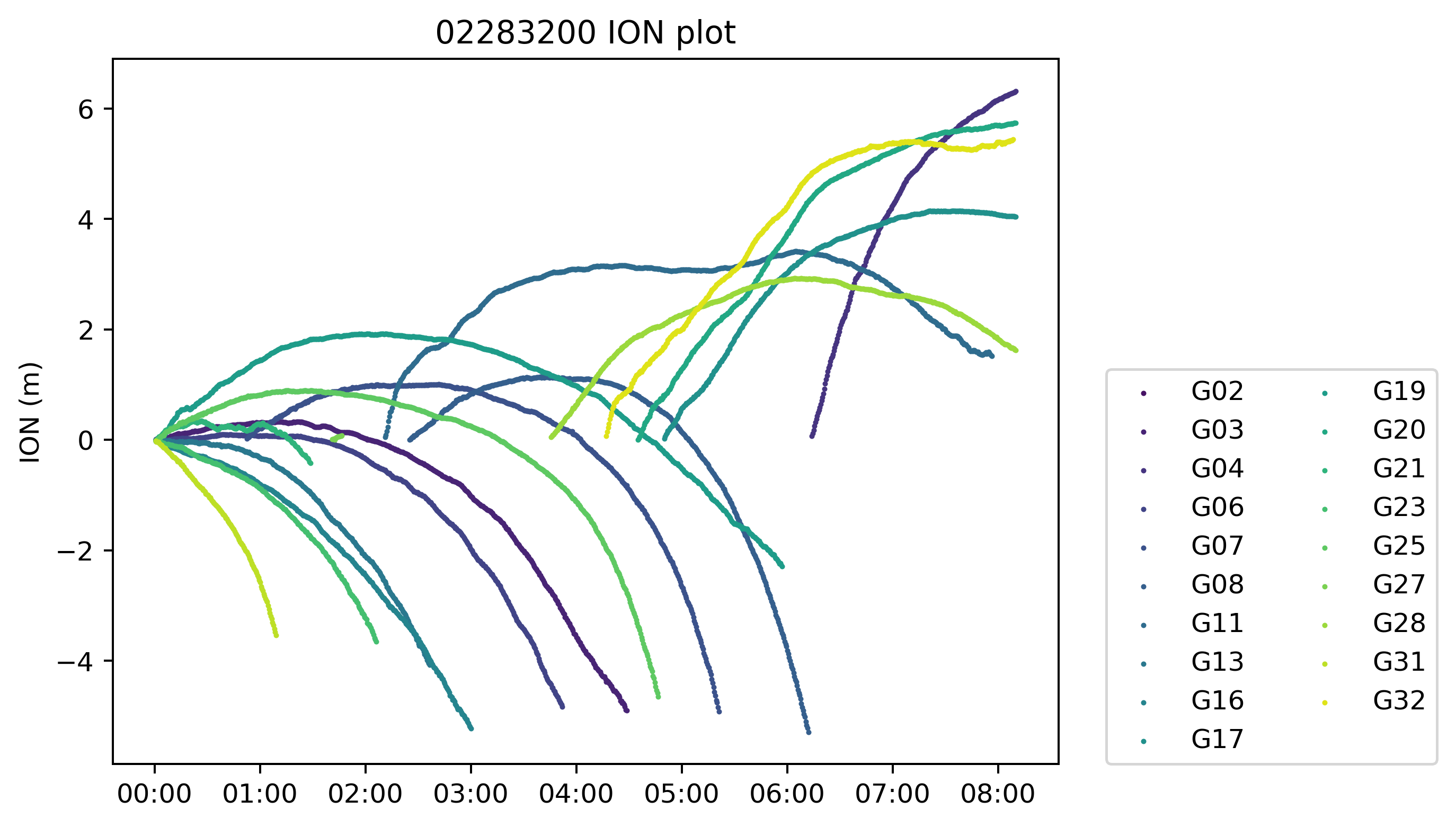

Supplement: Supplemental Information 3 [file peerj-cs-10-1800-s003.zip › 02283200ION_plot.png]

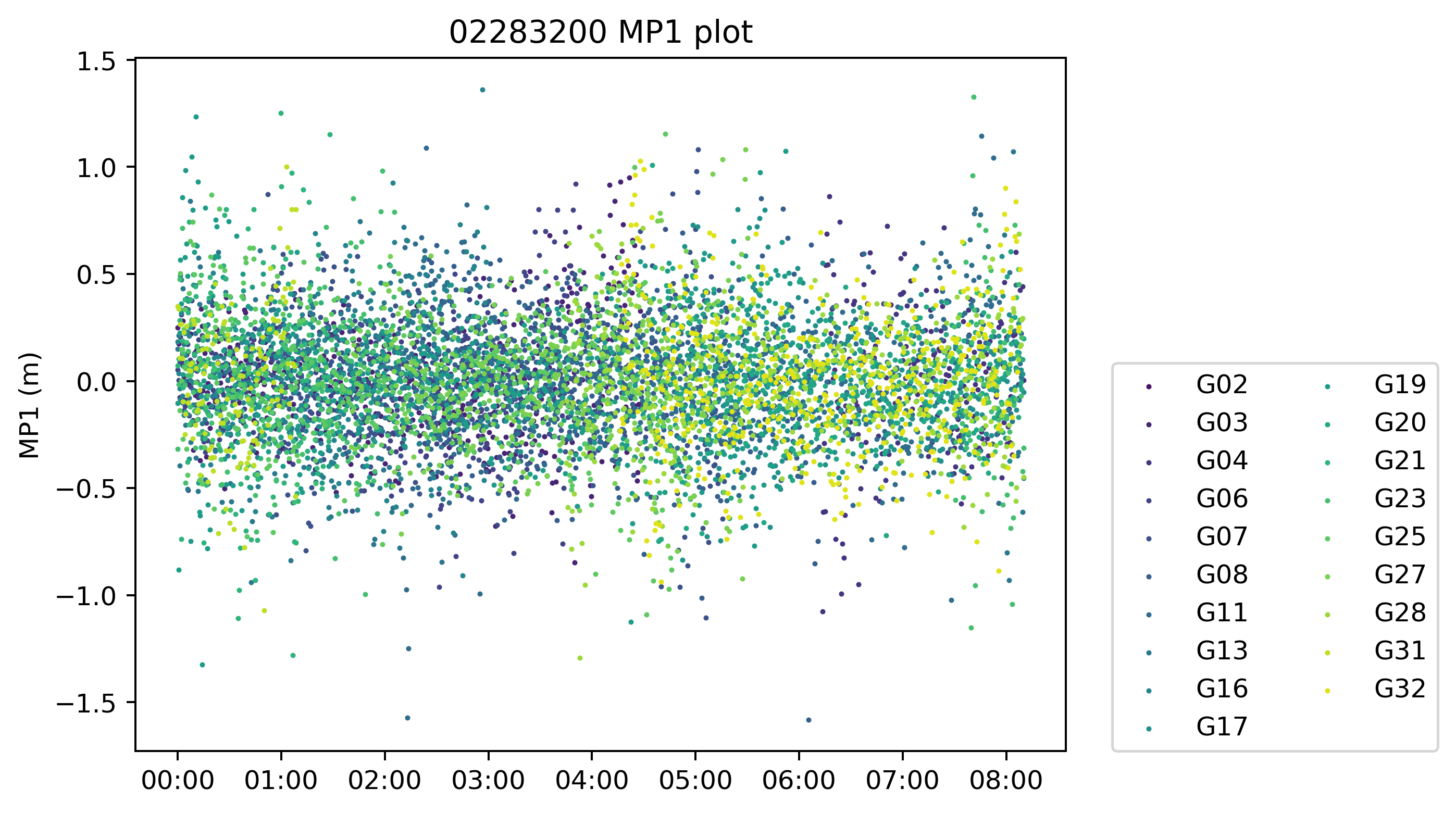

Supplement: Supplemental Information 3 [file peerj-cs-10-1800-s003.zip › 02283200MP1_plot.png]

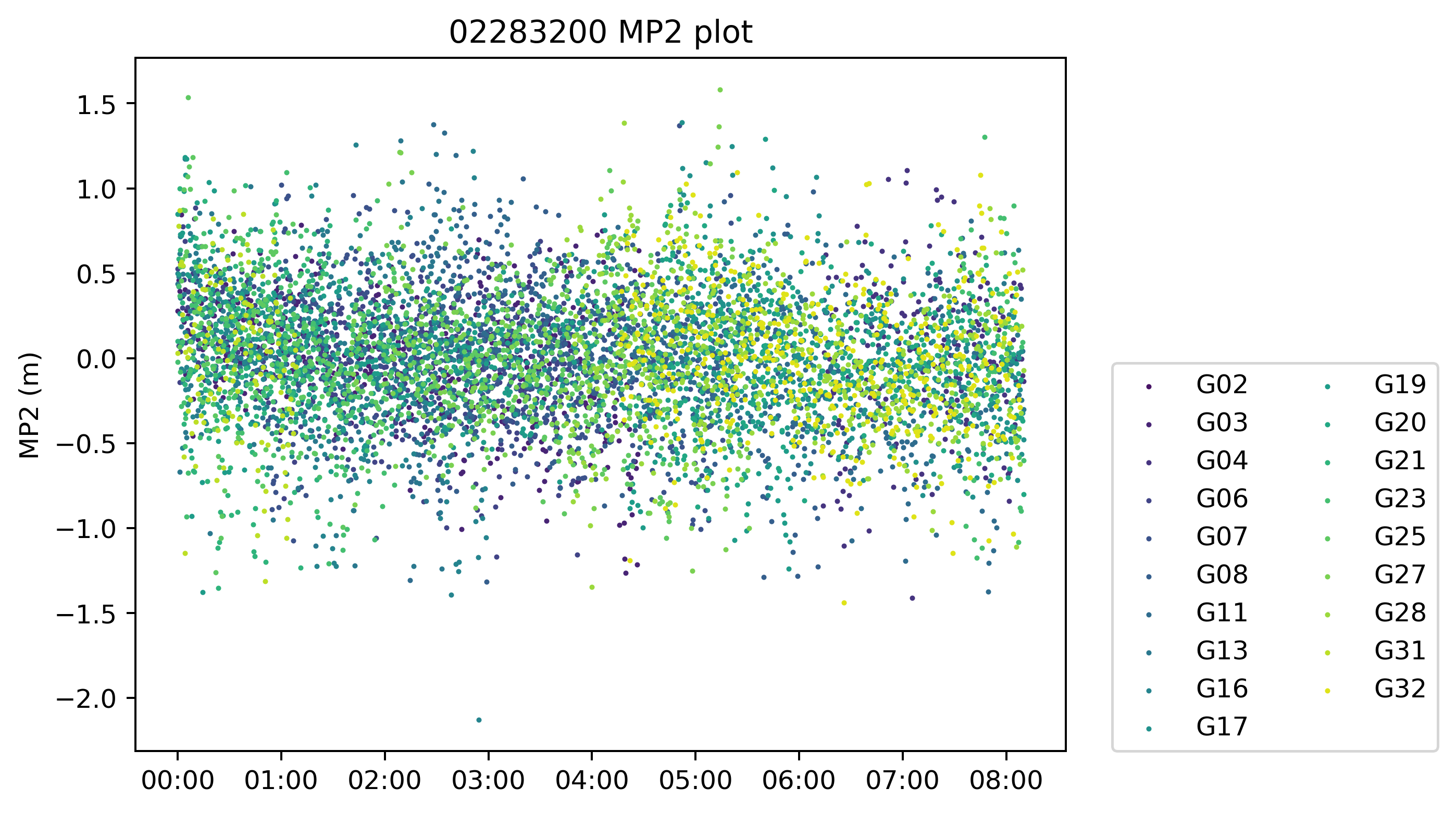

Supplement: Supplemental Information 3 [file peerj-cs-10-1800-s003.zip › 02283200MP2_plot.png]

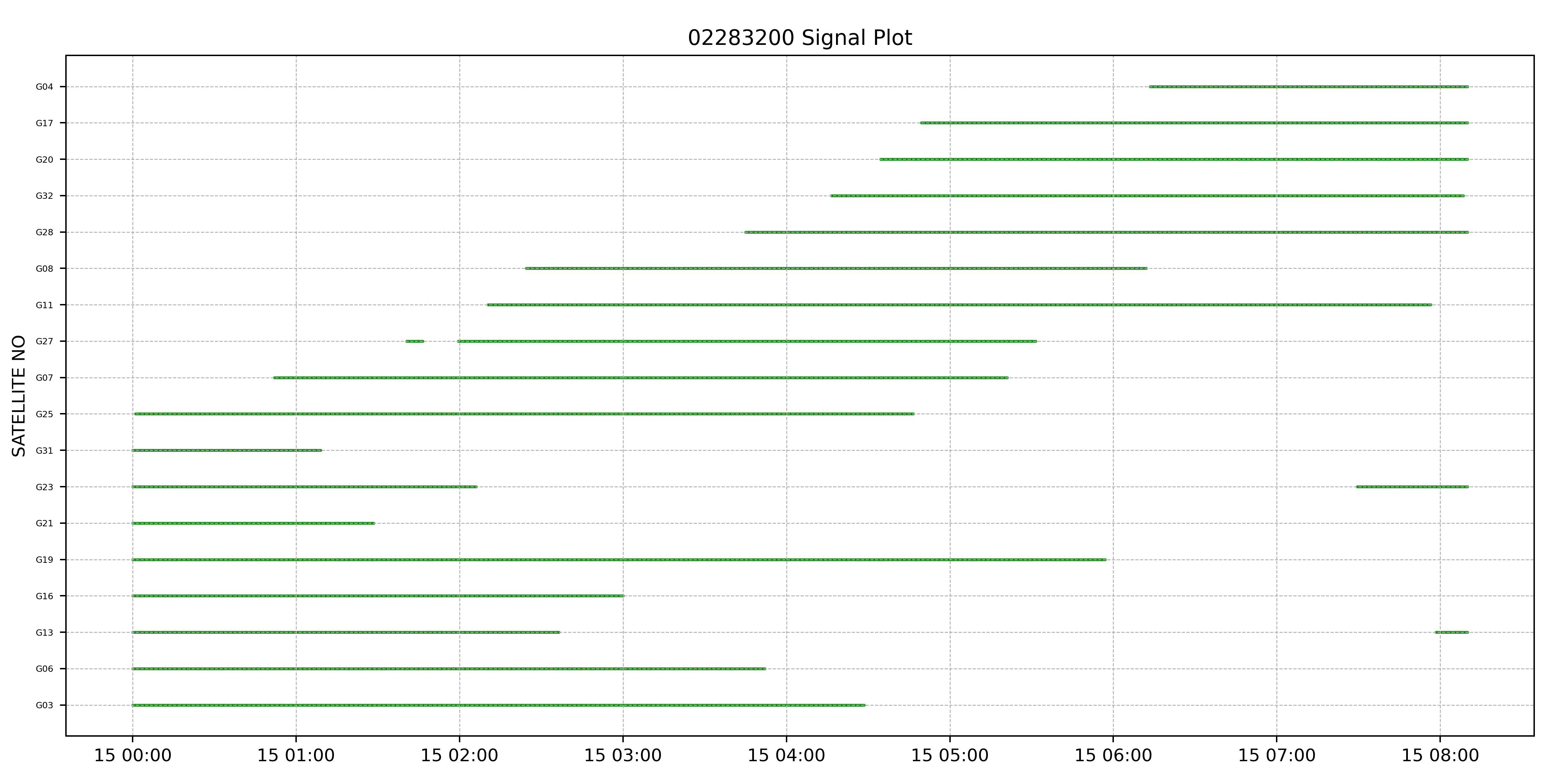

Supplement: Supplemental Information 3 [file peerj-cs-10-1800-s003.zip › 02283200SignalPlot.png]

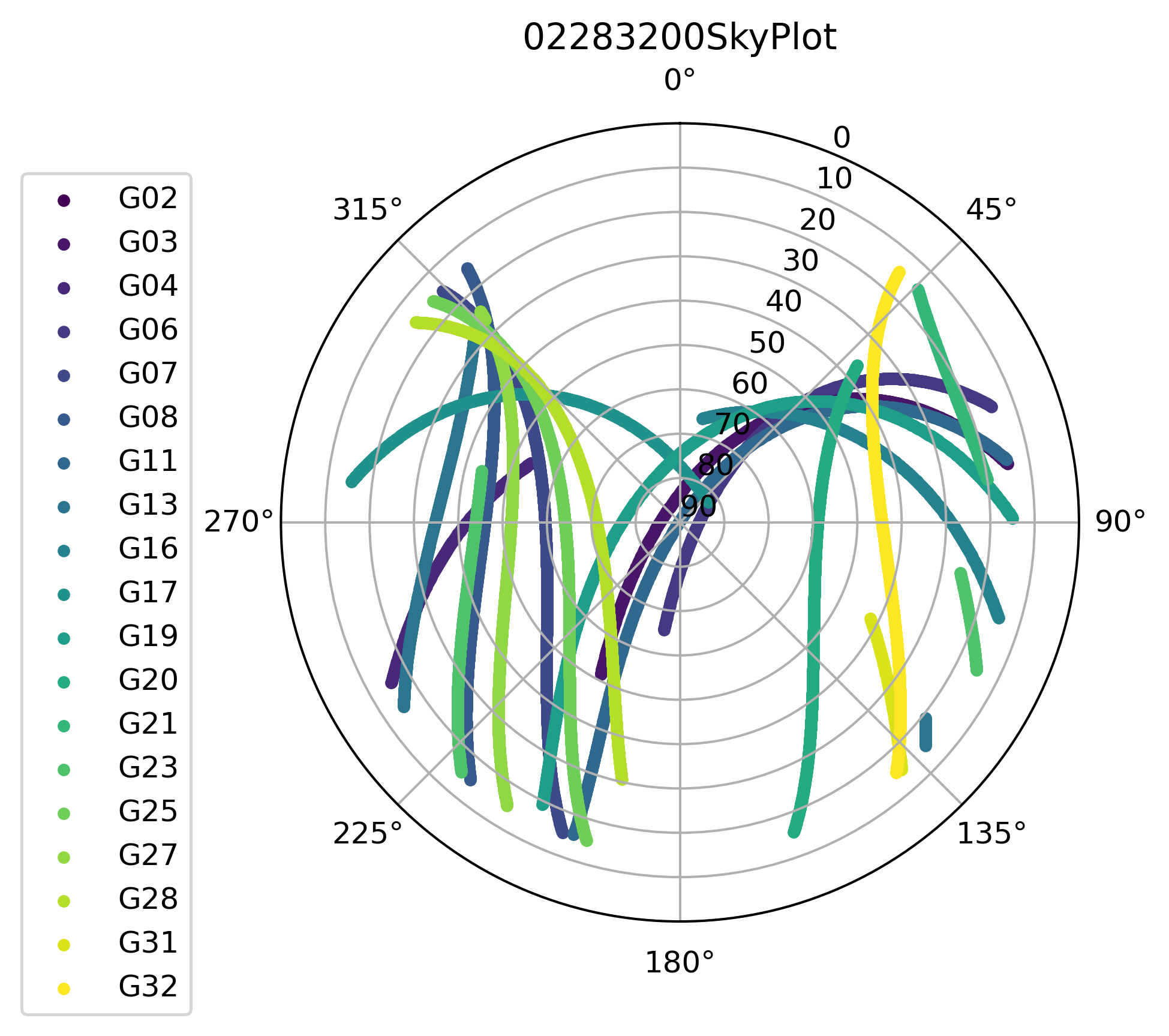

Supplement: Supplemental Information 3 [file peerj-cs-10-1800-s003.zip › 02283200skyplot.png]
