# Supplementary material for: PyRINEX: a new multi-purpose Python package for GNSS RINEX data
Source: PeerJ Comput Sci. 2024 Jan 16;10:e1800. doi: 10.7717/peerj-cs.1800 (PMC10803049; doi:10.7717/peerj-cs.1800)
Supplement: Supplemental Information 5 [file peerj-cs-10-1800-s005.pdf]

Install

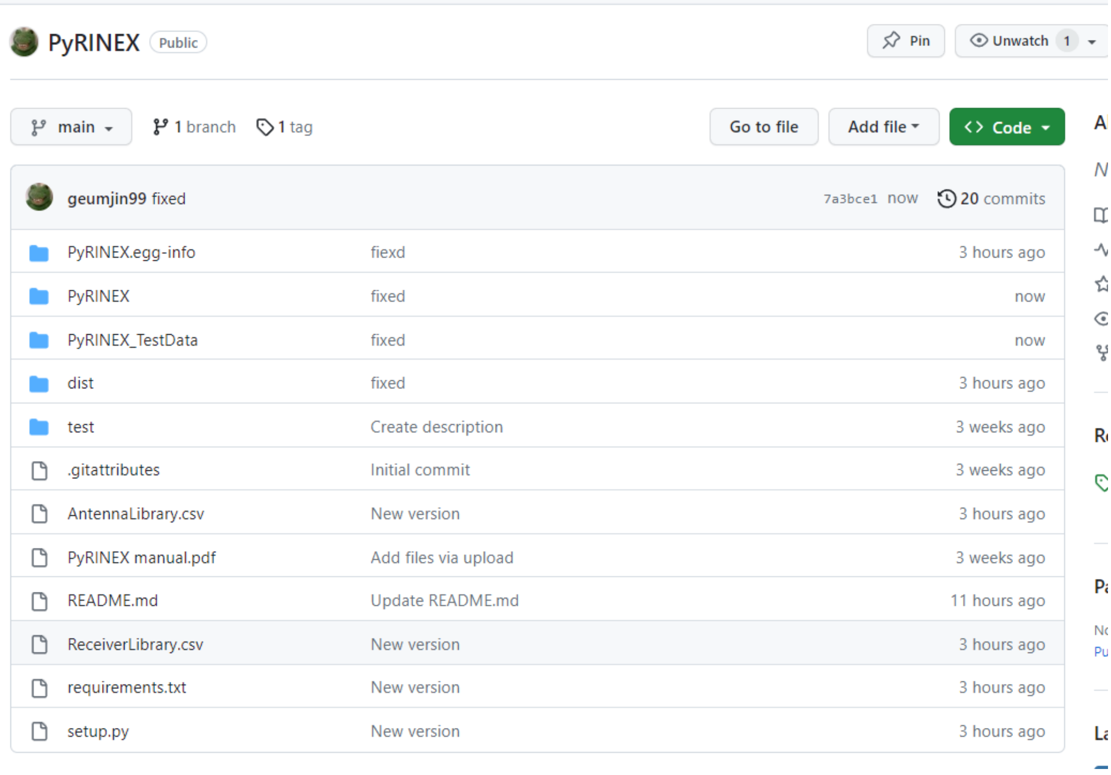

Figure 1 GitHub repository

The GitHub repository link is https://github.com/geumjin99/PyRINEX, you can download it or just clone it. The Test folder contains examples used in the paper.

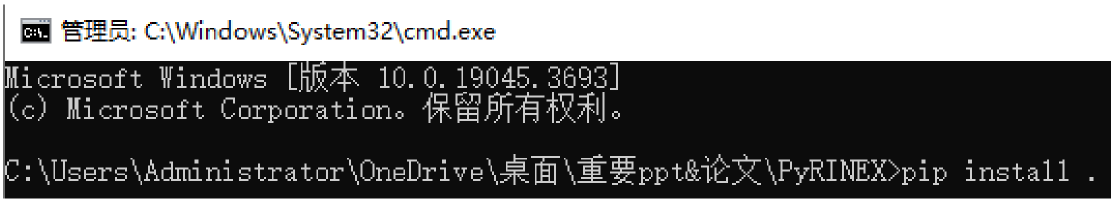

Figure 2 How to install

If you are downloading, clock CMD in the downloaded path and enter the command phrase as shown in Figure 2.

Please note that you may need to install the latest version of Visual Studio Build Tools. make sure that the "Desktop development with C++" workload is selected during installation, as some of the dependencies in Pandas may require a C++ compiler.

The PyRINEX software project (hereinafter referred to as the "Project") is released under the Apache Software License 2.0 (hereinafter referred to as the "License"). Anyone using this Project must comply with the terms of this License.

(If you want to quickly use PyRINEX for your research, you can skip these chapters and go straight to the last chapter, where we

illustrate the practical use of PyRINEX with the provided test data as an example.)

How to use PyRINEX

PyRINEX and other Python libraries such as matplotlib in the use of the method is not very different, import PyRINEX in IDE, and call the functions.

```
from PyRINEX import reader as rd
from PyRINEX import QulityCheck as qc
from PyRINEX import DataManagement as dm

rd.observations("C:\\Users\\Administrator\\OneDrive\\桌面\\suwn\\suwnall\\00013200.08o")
qc.QualityCheck("C:\\Users\\Administrator\\OneDrive\\桌面\\suwn\\suwnall\\00013200.08o")
dm.DataFinding("C:\\Users\\Administrator\\OneDrive\\桌面\\suwn", ["320"], ".08o")
```

Figure 3 Example of how to use PyRINEX

Figure 3 shows how PyRINEX is used. After importing PyRINEX's various modules, it can be easily called to realize its various functions.

In the next few sections we will specify the usage of the functions in each module and the form of their output results.

Reader

Table 1 Functions in Reader module

Functions	Parameter
oheader(opath)	observation file path
observations(opath)	observation file path
navigaions(opath)	navigation file path

Functions that can read RINEX data are provided in the Reader module, and they can read RINEX observation files and navigation files. They both require the path to the RINEX data to be entered as an argument and return a new json formatted data called LITE RINEX.

```
version 2
type G
receiver_type [3, 'TRIMBLE 5700        ']
antenna_type [4, 'TRM39105.00         ']
MARKER_NAME [8, 'BM01003012']
MARKER_NUMBER [9, 'BM01003012']
APPROX POSITION XYZ [-3173543.0196, 4134173.2686, 3666275.4904]
TIME OF FIRST OBS ['2013-8-12-8-10-30.0000000']
END OF HEADER 45
PRNS ['G01', 'G03', 'G06', 'G07', 'G08', 'G09', 'G11', 'G13', 'G14',
ObsTypes ['L1', 'C1', 'L2', 'P2', 'D1']
```

Figure 4 Output of the oheader function (If you wish to print that result in the IDE for review, use a statement like print(json.loads("your0010.23o")).)

Figure 4 shows the output of the oheader function. This contains some of the most important information in the header section in both categories, such as the version of the RINEX file, information about the type of observation recorded, etc. The second type of information is the marker name, receiver type, etc. The most important feature of this type of information is that it can be edited according to the user's needs, and it can be seen that this type of information is stored in a list, which is because the number of rows where these information are located is not fixed, so the line number is stored in the first item of the list for the purpose of modifying the information later on in the original file.

```
13 8 12 8 7 0.0110000 {'sat_num': 6, 'G01': {'L1': '   -860455.08606', 'C1': '  25055170.64006', 'L2': '
13 8 12 8 7 30.0110000 {'sat_num': 6, 'G01': {'L1': '   -772302.85207', 'C1': '  25071945.02207', 'L2': '
13 8 12 8 8 0.0110000 {'sat_num': 6, 'G01': {'L1': '   -683993.53907', 'C1': '  25088749.89007', 'L2': '
13 8 12 8 8 30.0110000 {'sat_num': 6, 'G01': {'L1': '   -595526.40207', 'C1': '  25105585.06907', 'L2': '
13 8 12 8 9 0.0110000 {'sat_num': 6, 'G01': {'L1': '   -506900.59406', 'C1': '  25122449.42906', 'L2': '
13 8 12 8 9 30.0110000 {'sat_num': 6, 'G01': {'L1': '   -418120.06306', 'C1': '  25139344.64006', 'L2': '
13 8 12 8 10 0.0110000 {'sat_num': 6, 'G01': {'L1': '   -329186.71106', 'C1': '  25156268.03006', 'L2': '
13 8 12 8 10 30.0110000 {'sat_num': 6, 'G01': {'L1': '   -240103.90206', 'C1': '  25173219.96806', 'L2':
```

Figure 5 Output of the observations function

As shown in figure 5, the same logic applies to the translation of the logged portion of the observations. PyRINEX provides the ability to translate RINEX observations and GPS navigation files into LITE RINEX format. This is extremely helpful for a number of studies that aim to process GNSS observation data.

```
G01 [{'EPOCH': '13  8 12  6  0  0.0', 'SV clock bias': '  .619990751147D-04', 'SV clock drift': '
G03 [{'EPOCH': '13  8 12  2  0  0.0', 'SV clock bias': '  .244840979576D-03', 'SV clock drift': '
G06 [{'EPOCH': '13  8 12  1 59 44.0', 'SV clock bias': '  .977194868028D-04', 'SV clock drift': '
G07 [{'EPOCH': '13  8 12  3 59 44.0', 'SV clock bias': '  .246392562985D-03', 'SV clock drift': '
G08 [{'EPOCH': '13  8 12  6  0  0.0', 'SV clock bias': '  .791111961007D-05', 'SV clock drift': '
G09 [{'EPOCH': '13  8 12  6  0  0.0', 'SV clock bias': '  .260732602328D-03', 'SV clock drift': '
G11 [{'EPOCH': '13  8 12  4  0  0.0', 'SV clock bias': ' -.409457832575D-03', 'SV clock drift': '
G13 [{'EPOCH': '13  8 12  1 59 44.0', 'SV clock bias': '  .909166410565D-04', 'SV clock drift': '
G14 [{'EPOCH': '13  8 11 23 59 28.0', 'SV clock bias': '  .218062195927D-03', 'SV clock drift': '
G16 [{'EPOCH': '13  8 12  0  0  0.0', 'SV clock bias': ' -.248799100518D-03', 'SV clock drift': '
G17 [{'EPOCH': '13  8 12  8  0  0.0', 'SV clock bias': ' -.104019418359D-04', 'SV clock drift': '
G19 [{'EPOCH': '13  8 12  1 59 44.0', 'SV clock bias': ' -.395882409066D-03', 'SV clock drift': '
G20 [{'EPOCH': '13  8 12  8  0  0.0', 'SV clock bias': '  .115176197141D-03', 'SV clock drift': '
G21 [{'EPOCH': '13  8 12  2  0  0.0', 'SV clock bias': ' -.313123222440D-03', 'SV clock drift': '
G23 [{'EPOCH': '13  8 12  2  0  0.0', 'SV clock bias': '  .583622604609D-04', 'SV clock drift': '
```

Figure 6 Output of the navigations function

Figure 6 shows the output of the navigations function, each satellite represented by a key will correspond to a list as a value, this is because it is possible for a satellite to be logged multiple times in the navigation file and the list will store them together.

DataManagement

```python
def DataFinding(root_path, keywordslist, RINEXextension):
    RINEXfiles = []
    for foldername, subfolders, filenames in os.walk(root_path):
        for filename in filenames:
            if all(keyword in filename for keyword in keywordslist) and filename.endswith(RINEXextension):
                file_path = os.path.join(foldername, filename)
                RINEXfiles.append(file_path)
    return RINEXfiles
```

Figure 7 DataFinding function in DataManagement module

As shown in figure 7, the DataFinding function implements the function of retrieving and filtering the RINEX data under a certain path. The function is implemented based on Python's os library, which is used to interact with the operating system, including file and directory operations. To use this function the user needs to enter the specified root directory, a list of keywords to filter its subfolders, and a extension representing the type of RINEX data file. After that, it will traverse all the files under the path, and then determine whether it meets the conditions, it is worth noting that even if the input extension is "08o", some files with "08O" as extension will still be output in the result list because the RINEX standard format does not specify the extension case.

```python
def DataCleaning(RINEX_FILES,ReceiverLibraryPath, AntennaLibraryPath, newfolder_root):
    fieldnames = ["origin path", "version", "station", "non English", "origin marker", "origin rec", "origin ant",
                  "new path", "marker", "longitude", "latitude", "rec type", "ant type"]
```

Figure 8 DataCleaning function in DataManagement module

As shown in figure 8, in DataCleaning function, you need to input the root path of the RINEX data that you want to be processed, the path of the ReceiverLibrary and AntennaLibrary, and the path that you want to output after data cleaning. The basic logic for PyRINEX to modify the four aforementioned errors is similar.

The DataCleaning function in DataManagement provide automatic errata for LITE RINEX after reading. It should be noted that for receiver type and antenna type corrections, the CSV files ReceiverLibrary and AntennaLibrary need to be read first, and PyRINEX will store the incorrect spelling and correct spelling as keys and values, respectively, as dictionaries in Python after reading them. After that, PyRINEX will check if there is a key in dictonray when it reads the corresponding line of the two contents, and if there is, it will replace it with the corresponding value, so that it can correct the specific contents in this way. The two CSV files can be freely edited by the user, which makes the processing of the data more customizable. CSV should like as shown in figure 9. Note that the line that records the antenna type in the RINEX data is required to record the radome type, but many RINEX data do not record this information, and not all programs that process RINEX data are required to read the radome type, but PyRINEX still modifies lines that do not have the radome information marked on them. However, PyRINEX will still modify RINEX data that do not have radome information marked on them by labeling them with "NONE" after the antenna type.

TRIMBLE 4000	?	TRIMBLE 4000S
TRIMBLE4700	?	TRIMBLE 4700
TRIMBLE5700	?	TRIMBLE 5700
TRIMBLE5800	?	TRIMBLE 5800
TRIMBLE5800II	?	TRIMBLE 5800
TPS GB1000	?	TPS GB-1000
TRIMBLENETR9	?	TRIMBLE NETR9

Figure 9 Example of ReceiverLibrary.csv/AntennaLirary.csv (The two CSV files available on Github were used in our research, and should be modified if you intend to apply them to the RINEX dataset you want to work with. In particular, if you need to work with a RINEX dataset that doesn't have a spelling error in the receiver and antenna type you should clear the first and third columns of the CSV files. However, the question mark in the second column must be saved.)

For the protection of raw data, the new RINEX file after data cleaning will be written to a specified new path, user needs to specify the root path of the output. After that, PyRINEX will use the mkdir function in the os library to create a new folder with the corresponding "doy" name and write it to it, and then when there are RINEX data observed on the same date that are cleansed by the data, they will also be written to this folder, which can help to organize a large amount of unorganized RINEX data. The reporter CSV file is shown in figure 10.

origin path	version	station	non l	origin n	origin rec	origin ant	new path	marker	longitude	latitude	rec type	ant type	filename
F:\gpsdata\01	2		yes	7182	TPS GB100(	TPSPG-A1	F:\RINEX07	7182	128.3502	37.56502	TPS GB-10(	TPSPG-A1	TRUE
F:\gpsdata\01	2		yes	7183	TPS GB100(	TPSPG-A1	F:\RINEX07	7183	128.2721	37.52085	TPS GB-10(	TPSPG-A1	TRUE
F:\gpsdata\01	2		yes	7174	TPS GB100(	TPSPG_A1	F:\RINEX07	7174	128.1715	37.72236	TPS GB-10(	TPSPG_A1	TRUE
F:\gpsdata\01	2		yes	7175	TPS GB100(	TPSPG_A1	F:\RINEX07	7175	128.0185	37.67045	TPS GB-10(	TPSPG_A1	TRUE
F:\gpsdata\01	2		yes	7171	TPS GB100(	TPSPG_A1	F:\RINEX07	7171	127.9244	37.68351	TPS GB-10(	TPSPG_A1	TRUE
F:\gpsdata\01	2		yes	7169	TPS GB100(	TPSPG_A1	F:\RINEX07	7169	127.9106	37.70805	TPS GB-10(	TPSPG_A1	TRUE
F:\gpsdata\01	2		yes	7179	TPS GB100(	TPSPG_A1	F:\RINEX07	7179	128.3068	37.74973	TPS GB-10(	TPSPG_A1	TRUE
F:\gpsdata\01	2		yes	7180	TPS GB100(	TPSPG-A1	F:\RINEX07	7180	128.4648	37.6714	TPS GB-10(	TPSPG-A1	TRUE
F:\gpsdata\01	2		yes	7184	TPS GB100(	TPSPG-A1	F:\RINEX07	7184	128.4596	37.49302	TPS GB-10(	TPSPG-A1	TRUE
F:\gpsdata\01	2		yes	7181	TPS GB100(	TPSPG-A1	F:\RINEX07	7181	128.4373	37.60671	TPS GB-10(	TPSPG-A1	TRUE
F:\gpsdata\01	2			169	TRIMBLE 57	TRM39105.(	F:\RINEX07	169	127.9087	37.70846	TRIMBLE 57	TRM39105.(	TRUE
F:\gpsdata\01	2			170	TRIMBLE 57	TRM39105.(	F:\RINEX07	170	127.8543	37.70401	TRIMBLE 57	TRM39105.(	TRUE
F:\gpsdata\01	2			171	TRIMBLE 57	TRM39105.(	F:\RINEX07	171	127.9263	37.64545	TRIMBLE 57	TRM39105.(	TRUE
F:\gpsdata\01	2			174	Unknown!	TRM39105.(	F:\RINEX07	174	128.1738	37.72003	TRIMBLE 57	TRM39105.(	TRUE

Figure 10 Example of reporter CSV file

QualityCheck

Table 2 Functions in QualityCheck module

Functions	Discription
plot (filename, title, gps serises, epochs, dataset, type, column, y_label = NONE, limit = "100")	Plot function for azi_ele(path), ION_MP(opath) and cycleslip(path)
SatelliteSignalPlot(path)	Output the signal plot
azi_ele(path)	Calculate the azimuth and elevation for GPS satellite
ION_MP(opath)	Calculate the multipath and ionospheric delay
cycleslip(path)	Calculate cycle slip
QualityCheck(path)	Perform all quality checks on a given RINEX data
batchQC(rootpath, keywords_list, extension)	Performs a quality check on RINEX data under a path, the principle is the same as the DataFinding function.

The plot function is responsible for visualizing the results of the quality check. It is worth noting, however, that if the user wishes to make changes to the function then the code for the parameters should not be changed, as these parameters are not actually entered by the user.

```
gps_prn = prns[1:]
name = os.path.basename(opath)
cloumn = math.ceil(len(prns)/18)
plot(opath[:-4] + "MP1_plot.png", name[:-4] + " MP1 plot", gps_prn, epochs, MP1MP2, 0, cloumn, y_label="MP1 (m)")
plot(opath[:-4] + "MP2_plot.png", name[:-4] + " MP2 plot", gps_prn, epochs, MP1MP2, 1, cloumn, y_label="MP2 (m)")
plot(opath[:-4] + "ION_plot.png", name[:-4] + " ION plot", gps_prn, epochs, MP1MP2, 2, cloumn, y_label="ION (m)")
plot(opath[:-4] + "IOD_plot.png", name[:-4] + " IOD plot", gps_prn, epochs, MP1MP2, 3, cloumn, y_label="IOD (m)")
[illegible]
```

Figure 11 Examples of using the plot function

As shown in Figure 11, this function will be called in the rest of the functions of the QUALITY CHECK, whose parameters are determined by these functions, such as COLUMN

determines how many columns the satellites should be written in the legend. Please note that in the plot function there is a default parameter is the upper and lower limits, the default is 100, this is because a lot of RINEX data quality check results are poor, there may be some "bad points" so that the final output is not easy to review, if you need to modify the parameter can be used to change the upper and lower limits of the value and enter what you want.

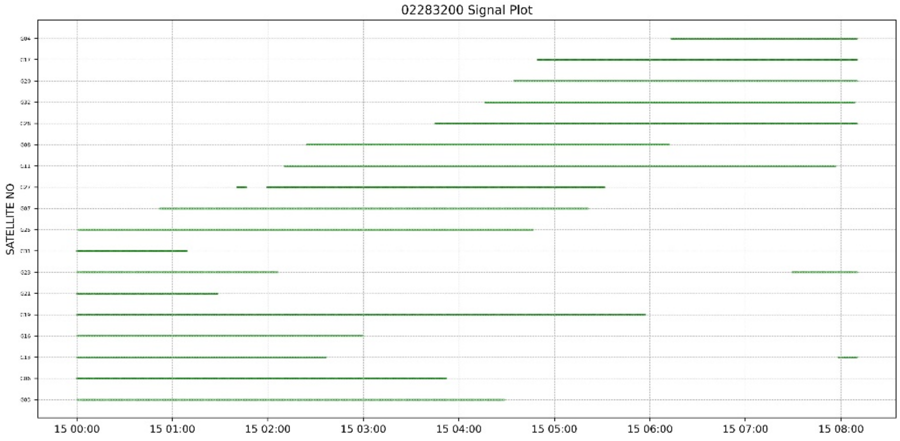

Figure 12 Examples output of the SatelliteSignalPlot function

In the SatelliteSignalPlot function, a visualization of the satellite models received by the receiver in each time slot is provided. The function outputs a schematic diagram, which allows the user to visualize the type of satellites received during each time period and, more importantly, to know which satellites have had interruptions in the reception of their signals, which means that it is possible that poor observing conditions have triggered difficulties in the reception of the signals. Figure 12 shows schematic output of the SatelliteSignalPlot function.

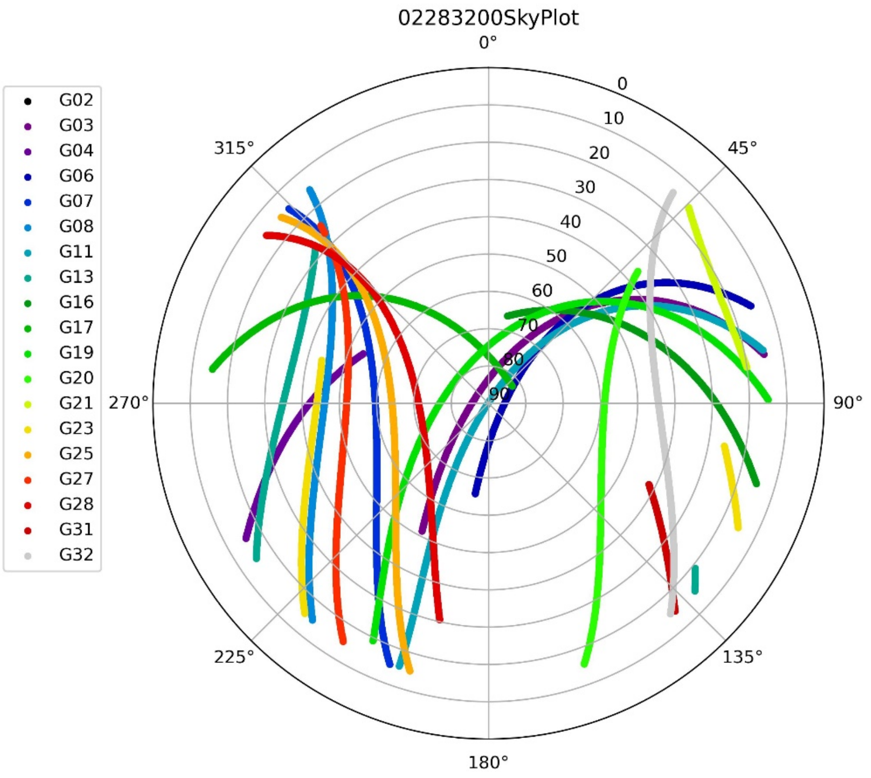

Figure 13 Examples output of the azi_ele function

When using the azi_ele function, you need to make sure that the RINEX data is internally logged with GPS satellite data and that the navigation file and the observation file are under the same path. The output of azi_ele function as shown in figure 13.

epoch	G01	G03	G06	G07	G08	G09	G11	G13	G14
13 8 12 0 0 30. 0010000									[2. 7231193335204305, 0. 6197214451251352]
13 8 12 0 1 0. 0010000									[2. 7244568001278826, 0. 6155936610163916]
13 8 12 0 1 30. 0010000									[2. 7257818460444057, 0. 6114685133291546]
13 8 12 0 2 0. 0010000									[2. 7270945500551527, 0. 6073460320514418]
13 8 12 0 2 30. 0010000									[2. 728394989348543, 0. 6032262469118151]
13 8 12 0 3 0. 0010000			[3. 3499638990221654, 0. 3854352735613723]						[2. 729683239548119, 0. 5991091873825859]
13 8 12 0 3 30. 0010000			[3. 349890485774989, 0. 3893519244181958]						[2. 730959374743607, 0. 5949948826829812]
13 8 12 0 4 0. 0010000			[3. 3498231289198404, 0. 3932741979847059]						[2. 732223467521178, 0. 5908833617822565]
13 8 12 0 4 30. 0010000			[3. 3497618094959027, 0. 39720207952891445]						[2. 7334755889929663, 0. 5867746534027819]
13 8 12 0 5 0. 0010000			[3. 349706508559498, 0. 4011355542435031]						[2. 734715808825822, 0. 5826687860230527]
13 8 12 0 5 30. 0010000			[3. 3496572071828656, 0. 4050746072441165]						[2. 735944195269368, 0. 5785657878807001]
13 8 12 0 6 0. 0010000			[3. 349613886452906, 0. 40901922356767506]						[2. 7371608151833433, 0. 5744656869754263]
13 8 12 0 6 30. 0010000			[3. 349576527469898, 0. 41296938817066836]						[2. 7383657340642706, 0. 5703685110719238]
13 8 12 0 7 0. 0010000			[3. 349545111346179, 0. 416925085927475]						[2. 7395590160714662, 0. 566274287702752]
13 8 12 0 7 30. 0010000			[3. 3495196192047985, 0. 42088630162866625]						[2. 7407407240524027, 0. 5621830441711745]
13 8 12 0 8 0. 0010000			[3. 349500032178138, 0. 424853019979318]						[2. 74191091956745, 0. 5580948075539658]
13 8 12 0 8 30. 0010000			[3. 349486331406492, 0. 42882522559733355]						[2. 7430696629140137, 0. 5540096047041884]
13 8 12 0 9 0. 0010000			[3. 349478498036628, 0. 4328029030117548]						[2. 7442170131500756, 0. 549927462253933]
13 8 12 0 9 30. 0010000			[3. 3494765132202984, 0. 43678603666109006]						[2. 745353028117168, 0. 545848406617031]
13 8 12 0 10 0. 0010000			[3. 3494803581127215, 0. 440774610891639]						[2. 7464777644627825, 0. 5417724639917361]
13 8 12 0 10 30. 0010000			[3. 3494900138710326, 0. 44476860995582074]						[2. 747591277662249, 0. 5376996603633828]
13 8 12 0 11 0. 0010000			[3. 349505461652691, 0. 4487680180105107]						[2. 7486936220400704, 0. 5336300215070113]
13 8 12 0 11 30. 0010000			[3. 3495266826138477, 0. 45277281911538053]						[2. 7497848507907543, 0. 5295635729899685]
13 8 12 0 12 0. 0010000			[3. 349553657907684, 0. 4567829972312356]						[2. 750865015999147, 0. 5255003401744867]

Figure 14 Examples output CSV file of the azi_ele function

The CSV file output together with the azi_ele function is shown in Figure 14, with the first data in each cell representing the azimuth and the second data representing the elevation angle (It's all in arcs).

Note that both the CSV file and the charts are generated in the same path as the original RINEX data, which is the same for all subsequent quality check related functions.

```
epoch 13 8 12 0 0 30.0010000
G14 [2.7231193335204305, 0.6197214451251352]
G16 [4.381956806587844, 0.7518445094435549]
G29 [0.9795007528779629, 0.5891365465569922]
G31 [0.7771883872963753, 1.1030195802322884]
G32 [4.4978594096955, 0.3914340658522126]
```

Figure 15 Examples output Numpy array of the ION_MP function

At the same time, the output of this function is written to a list, where each element of the list is a dictionary as shown in Figure 15.

	G01	G02	G03	G04
EPOCH1	[0, 0, 0, 0]	[MP1, MP2, ION, IOD]	[MP1, MP2, ION, IOD]	[0, 0, 0, 0]
EPOCH2	[MP1, MP2, ION, IOD]	[MP1, MP2, ION, IOD]	[0, 0, 0, 0]	[MP1, MP2, ION, IOD]
EPOCH3	[0, 0, 0, 0]	[0, 0, 0, 0]	[0, 0, 0, 0]	[MP1, MP2, ION, IOD]
EPOCH4	[MP1, MP2, ION, IOD]	[MP1, MP2, ION, IOD]	[MP1, MP2, ION, IOD]	[MP1, MP2, ION, IOD]

Figure 16 Examples output Numpy array of the ION_MP function

For the two functions ION_MP and cycleslip, their most important feature is that they will output a Numpy array in addition to the CSV file and the graph, which can be convenient for the user to carry out subsequent calculations. The ordering of the output data in the third dimension of this three-dimensional array is shown in Figure 13 (The output of cycleslip has an index of 3 in the third dimension.).

The results of MP1 and MP2, as well as ION and IOD, are output to four different plots, but then written two by two to the same CSV file, where the order of precedence in each cell is the same as in Figure 14.

The cycleslip function is identical.

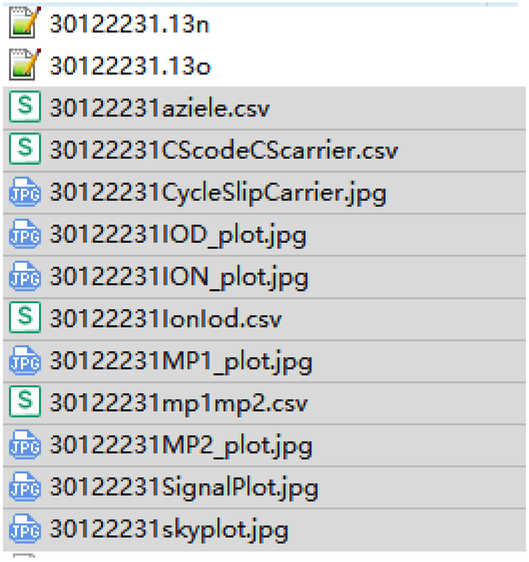

Figure 16 Example of output after using the QualityCheck function.

When using the QualityCheck function, it automatically runs all of the above quality check calculations at once and produces the result file shown in Figure 16 under the same path. However, if the navigation file does not exist in the same path, PyRINEX will skip the azi_ele function and only execute other functions.

The operation mechanism of batchQC function is similar to that of DataFinding function, users only need to input keywords to batch process specific RINEX data under a folder.

Practical use cases of PyRINEX

The data we use as examples in this chapter can be found in the Github repository, and you can get him whether you rely on gitclone or just download the zip.

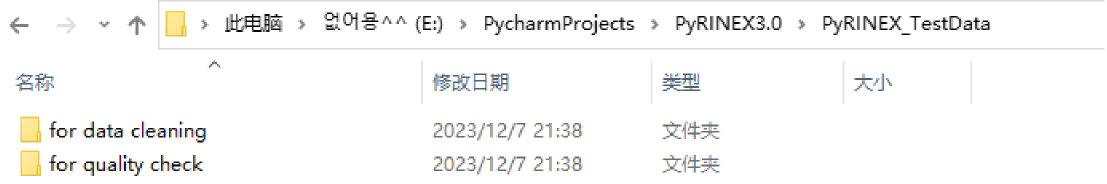

Figure 17 PyRINEX_TestData folder

As shown in Figure 17, there are two subfolders under this folder, which contain example data for DATA CLEANING and QUALITY CHECK, respectively. The subfolder also includes two folders, test data and results, which contain test data and runtime results, respectively.

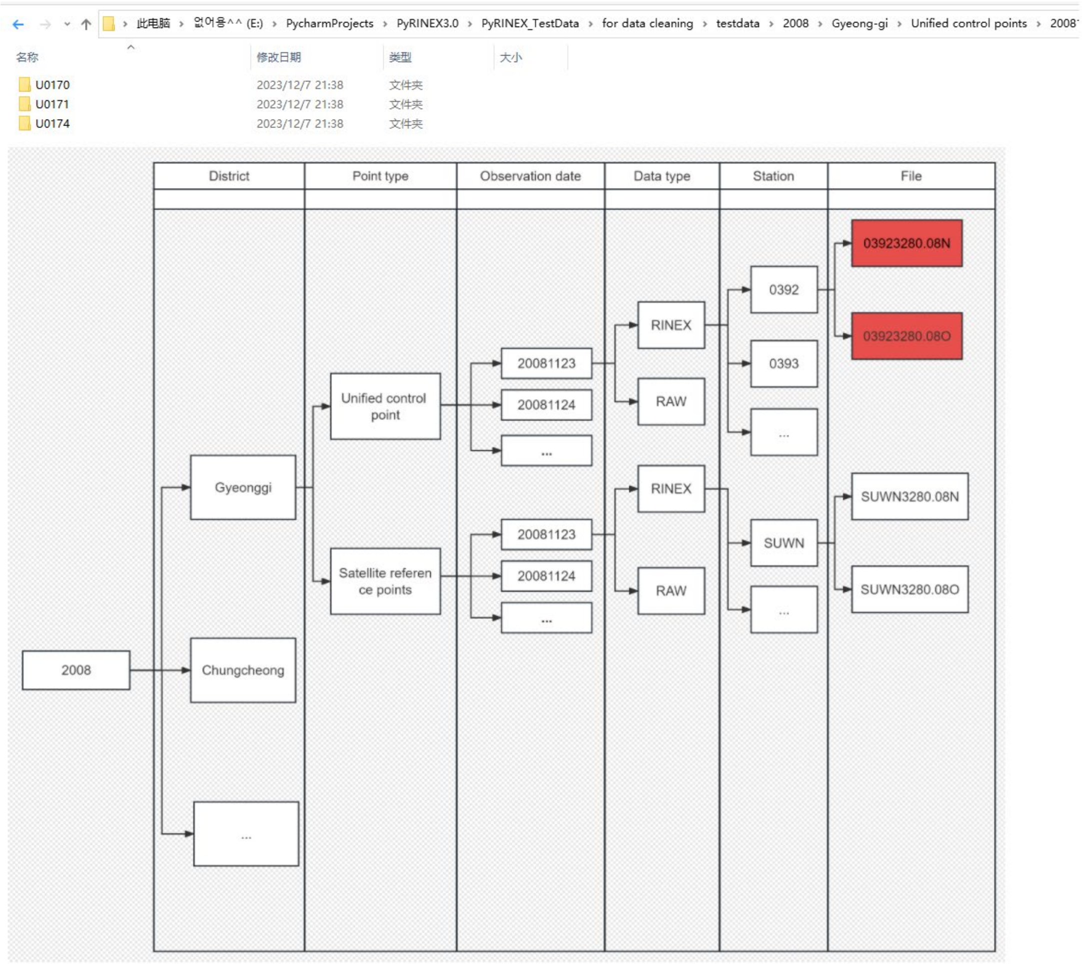

Figure 18 Diagram of the structure of the data cleaning test folder

As shown in Figure 18, the structure of the folder for data cleaning tests is the same as the dataset used in the experimental part of the paper, which we used to demonstrate how to perform data cleaning.

The goal is to write all the RINEX files in the Gyeong-gi folder under the path of the unified control points into a clearer path, and at the same time to fix the formatting errors within them.

```
from PyRINEX import reader as rd
from PyRINEX import QulityCheck as qc
from PyRINEX import DataManagement as dm

rootpath = "C:\\Users\\Administrator\\OneDrive\\桌面\\重要ppt&论文\\PyRINEX_TestData\\for data cleaning\\testdata"
keywordslist = ["Gyeong-gi", "Unified control points"]
ReceiverLibraryPath = "C:\\Users\\Administrator\\OneDrive\\桌面\\重要ppt&论文\\PyRINEX\\ReceiverLibrary.csv"
AntennaLibraryPath = "C:\\Users\\Administrator\\OneDrive\\桌面\\重要ppt&论文\\PyRINEX\\AntennaLibrary.csv"
newfolder_root = "C:\\Users\\Administrator\\OneDrive\\桌面\\重要ppt&论文\\PyRINEX_TestData\\for data cleaning\\result"
dm.DataCleaning(dm.DataFinding(rootpath, keywordslist, "08O"),
                ReceiverLibraryPath, AntennaLibraryPath, newfolder_root, radome=True)
```

Figure 19 Example of data cleaning

As shown in Figure 19, we performed data cleaning using the DataCleaning function. We passed it five parameters in order: the root path where the data is located, a list of keywords, the ReceiverLibrary.csv file path, the AntennaLibrary.csv file path, and the new folder path.

Please note that if you have opened our AntennaLibrary.csv file you will find that it is empty, this is because in our test dataset we didn't find any misspellings of the antenna type, so we have just filled in the antenna radome as 'NONE! This is because we did not find any

misspelling of antenna type in our test dataset, so we just complete the antenna radome to 'NONE', if you want to apply it to your own dataset, please modify AntennaLibrary.csv file.

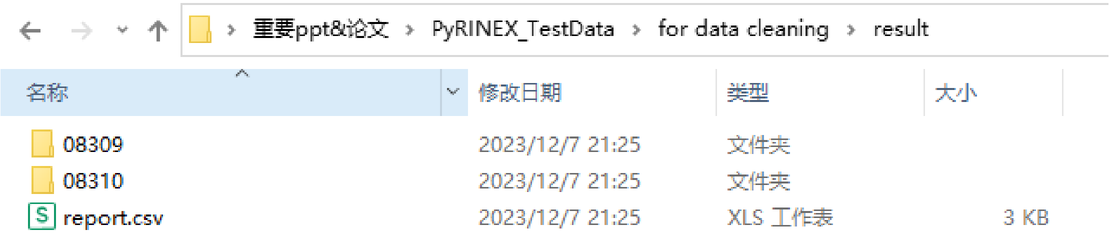

Figure 20 Example of running results

The result of the run is shown in Figure 20, where the target RINEX data are written under the corresponding new path, and the reporter is also generated in that path.

origin path	version	non English	origin ma	origin re	origin an	new path	marker	longitude	latitude	rec type	ant type	
C:\Users\Admini:	2		170	TRIMBLE 5	TRM39105.	C:\Users\	170	127.8543	37.70401	TRIMBLE 5	TRM39105.00	NONE
C:\Users\Admini:	2		171	TRIMBLE 5	TRM39105.	C:\Users\	171	127.9263	37.64545	TRIMBLE 5	TRM39105.00	NONE
C:\Users\Admini:	2		174	Unknown!	TRM39105.	C:\Users\	174	128.1738	37.72003	TRIMBLE 5	TRM39105.00	NONE
C:\Users\Admini:	2	Non-English characters present	CH06	TPS GB100	PG-A1	C:\Users\CH06		128.1378	37.51911	TPS GB-10	PG-A1	NONE
C:\Users\Admini:	2	Non-English characters present	PA65	TPS GB100	PG-A1	C:\Users\PA65		128.3904	37.36168	TPS GB-10	PG-A1	NONE

Figure 21 Example of reporter (The number "170" in marker is because CSV files can't write numbers as strings, so "0170" is automatically changed to 170, but this is not the case with RINEX data.)

As shown in Figure 21, the original and new paths of the RINEX data are recorded in the reporter, and the information of the old and new RINEX data is compared in detail inside.

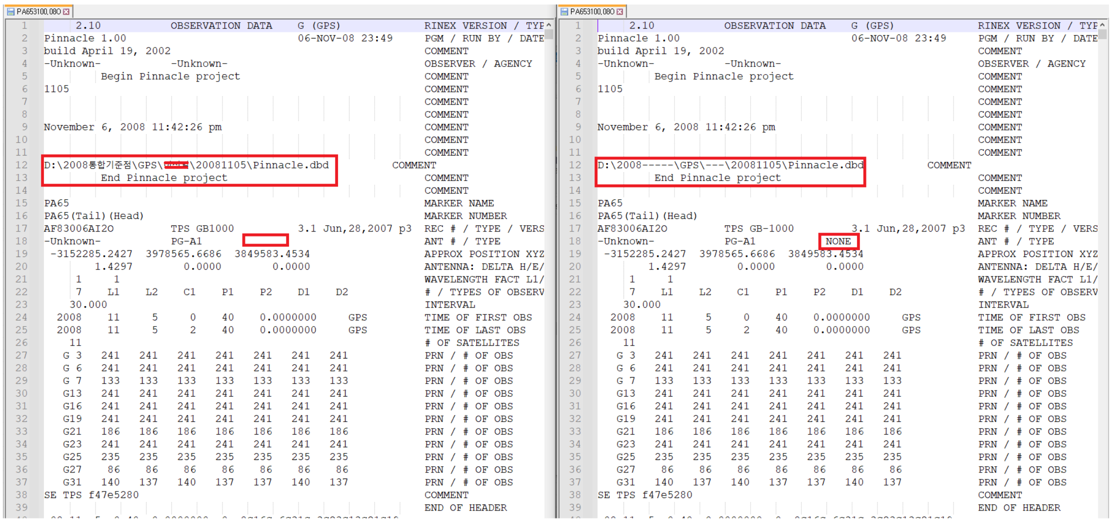

Figure 22 Results of formatting corrections

The format within the RINEX data was also corrected as shown in Figure 22. Some non-English characters have been replaced and the missing antenna radome has been patched.

Next we have a demonstration of QUALITY CHECK.

重要ppt&论文 › PyRINEX_TestData › for quality check › testdata

名称	修改日期	类型	大小
00013200.08n	2023/3/29 19:13	08N 文件	39 KB
00013200.08o	2023/3/29 19:13	08O 文件	567 KB
02213200.08n	2023/3/29 19:13	08N 文件	39 KB
02213200.08o	2023/3/30 10:04	08O 文件	564 KB
02283200.08n	2023/3/29 19:13	08N 文件	39 KB
02283200.08o	2023/3/29 19:13	08O 文件	564 KB
02303200.08n	2023/3/29 19:13	08N 文件	39 KB
02303200.08o	2023/3/29 19:13	08O 文件	566 KB
02313200.08n	2023/3/29 19:13	08N 文件	37 KB
02313200.08o	2023/3/29 19:13	08O 文件	526 KB
02913200.08n	2023/3/29 19:13	08N 文件	36 KB
02913200.08o	2023/3/29 19:13	08O 文件	527 KB
72213200.08n	2023/3/29 19:13	08N 文件	23 KB
72213200.08o	2023/3/29 19:13	08O 文件	264 KB
72303200.08n	2023/3/29 19:13	08N 文件	15 KB
72303200.08o	2023/3/29 19:13	08O 文件	148 KB
72913200.08n	2023/3/29 19:13	08N 文件	24 KB
72913200.08o	2023/3/29 19:13	08O 文件	257 KB
suwn3200.08o	2023/11/6 18:29	08O 文件	747 KB

Figure 23 "for quality check" folder and the data it contains

As shown in Figure 23, the data we provide is exactly the same data used for the trustworthiness evaluation of the quality check function of PyRINEX used in the paper.

```
from PyRINEX import reader as rd
from PyRINEX import QulityCheck as qc
from PyRINEX import DataManagement as dm


rootpath1 = "C:\\Users\\Administrator\\OneDrive\\桌面\\重要ppt&论文\\PyRINEX_TestData\\for quality check\\testdata"

qc.batchQC(rootpath1, [], ".08o")
```

Figure 24 Example of a batch quality check

As shown in Figure 24, we use the batchQC function to perform a quality check, which is used by entering the root path where the RINEX data that needs to be processed is located, and the path needs to contain a list of keywords (in this case, an empty list), as well as the suffix name of the RINEX data that is being processed.

00013200.08n	2023/3/29 19:13	08N 文件	39 KB
00013200.08o	2023/3/29 19:13	08O 文件	567 KB
00013200CScarrier.csv	2023/12/7 21:33	XLS 工作表	188 KB
00013200CycleSlipCarrier.png	2023/12/7 21:33	WPS.PIC.png	467 KB
00013200IOD_plot.png	2023/12/7 21:33	WPS.PIC.png	682 KB
00013200ION_plot.png	2023/12/7 21:33	WPS.PIC.png	339 KB
00013200IonIod.csv	2023/12/7 21:33	XLS 工作表	384 KB
00013200MP1_plot.png	2023/12/7 21:33	WPS.PIC.png	672 KB
00013200mp1mp2.csv	2023/12/7 21:33	XLS 工作表	384 KB
00013200MP2_plot.png	2023/12/7 21:33	WPS.PIC.png	785 KB
00013200SignalPlot.jpg	2023/12/7 21:33	JPG 图片文件	984 KB
02213200.08n	2023/3/29 19:13	08N 文件	39 KB
02213200.08o	2023/3/30 10:04	08O 文件	564 KB
02213200CScarrier.csv	2023/12/7 21:33	XLS 工作表	181 KB
02213200CycleSlipCarrier.png	2023/12/7 21:33	WPS.PIC.png	528 KB
02213200IOD_plot.png	2023/12/7 21:34	WPS.PIC.png	292 KB
02213200ION_plot.png	2023/12/7 21:34	WPS.PIC.png	541 KB
02213200IonIod.csv	2023/12/7 21:33	XLS 工作表	380 KB
02213200MP1_plot.png	2023/12/7 21:33	WPS.PIC.png	1,098 KB
02213200mp1mp2.csv	2023/12/7 21:33	XLS 工作表	379 KB
02213200MP2_plot.png	2023/12/7 21:34	WPS.PIC.png	1,095 KB
02213200SignalPlot.jpg	2023/12/7 21:34	JPG 图片文件	982 KB
02283200.08n	2023/3/29 19:13	08N 文件	39 KB
02283200.08o	2023/3/29 19:13	08O 文件	564 KB
02283200CScarrier.csv	2023/12/7 21:34	XLS 工作表	185 KB
02283200CycleSlipCarrier.png	2023/12/7 21:34	WPS.PIC.png	1,043 KB
02283200IOD_plot.png	2023/12/7 21:34	WPS.PIC.png	1,014 KB
02283200ION_plot.png	2023/12/7 21:34	WPS.PIC.png	521 KB
02283200IonIod.csv	2023/12/7 21:34	XLS 工作表	382 KB
02283200MP1_plot.png	2023/12/7 21:34	WPS.PIC.png	1,111 KB
02283200mp1mp2.csv	2023/12/7 21:34	XLS 工作表	382 KB
02283200MP2_plot.png	2023/12/7 21:34	WPS.PIC.png	1,130 KB
02283200SignalPlot.jpg	2023/12/7 21:34	JPG 图片文件	983 KB

Figure 25 The output of the batcQC run

As shown in Figure 25, the report files corresponding to the quality check results for each RINEX data after the run were written under the same path, including schematic and CSV files.
